# Coordinated Morphogenetic Mechanisms Shape the Vertebrate Eye

**DOI:** 10.3389/fnins.2017.00721

**Published:** 2017-12-20

**Authors:** Juan-Ramon Martinez-Morales, Florencia Cavodeassi, Paola Bovolenta

**Affiliations:** ^1^Centro Andaluz de Biología del Desarrollo (CSIC/UPO/JA), Seville, Spain; ^2^Centro de Biología Molecular Severo Ochoa, (CSIC/UAM), Madrid, Spain; ^3^CIBERER, ISCIII, Madrid, Spain

**Keywords:** morphogenesis, patterning, eye development, vertebrates, cell movement, retina pigment epithelim, cell shape

## Abstract

The molecular bases of vertebrate eye formation have been extensively investigated during the past 20 years. This has resulted in the definition of the backbone of the gene regulatory networks controlling the different steps of eye development and has further highlighted a substantial conservation of these networks among vertebrates. Yet, the precise morphogenetic events allowing the formation of the optic cup from a small group of cells within the anterior neural plate are still poorly understood. It is also unclear if the morphogenetic events leading to eyes of very similar shape are indeed comparable among all vertebrates or if there are any species-specific peculiarities. Improved imaging techniques have enabled to follow how the eye forms in living embryos of a few vertebrate models, whereas the development of organoid cultures has provided fascinating tools to recapitulate tissue morphogenesis of other less accessible species. Here, we will discuss what these advances have taught us about eye morphogenesis, underscoring possible similarities and differences among vertebrates. We will also discuss the contribution of cell shape changes to this process and how morphogenetic and patterning mechanisms integrate to assemble the final architecture of the eye.

The development of the vertebrate eye has attracted the interest of classical embryologists for more than a century and that of modern developmental geneticists for the past two decades (Spemann, 1901; Chow and Lang, 2001). This continuous interest is possibly linked to several of it characteristics. For example, the eye is much more accessible to experimental manipulations than the rest of the Central Nervous System (CNS); it is also an ideal model to study signaling and inductive events, as its formation involves the interaction among different tissues including the neural and non-neural ectoderm, the axial meso-endoderm, and the periocular mesenchyme (Fuhrmann, 2010; Sinn and Wittbrodt, 2013; Bazin-Lopez et al., 2015). The multi-branched evolutionary history behind the emergence of visual organs from an ancestral prototypic eye has also attracted the attention of researchers in the evo-devo field, converting the eye to an excellent tool for morphological and molecular evolutionary studies (Arendt, 2003; Letelier et al., 2017). The latter have helped to understand the logic of their gene regulatory networks and have established that vertebrate eye formation depends on the reiterative use of a core set of regulatory molecules highly conserved among invertebrates and vertebrates (Fuhrmann, 2010; Beccari et al., 2013). These gene regulatory networks are described in several comprehensive reviews to which the readers are referred to (Chow and Lang, 2001; Martinez-Morales et al., 2004; Adler and Canto-Soler, 2007; Fuhrmann, 2010; Cavodeassi and Houart, 2012; Beccari et al., 2013; Amram et al., 2017). Old morphological studies based on static images together with more recent molecular studies have also established that eye formation starts with the specification of eye field within the anterior neural plate (ANP), followed by their lateral protrusion to form the optic vesicles, and the infolding of the vesicles into bi-layered optic cups (Hilfer, 1983; Schmitt and Dowling, 1994; Li et al., 2000; Kwan et al., 2012).

## From a flat eye field to a tri-dimensional optic vesicle

The rough description of eye morphogenesis described above has been highly refined in the recent years. Technical advances in imaging procedures coupled with the use of transparent fish embryos, such as those of zebrafish and medaka fishes, have indeed provided a better grasp of the cell choreography that organizes eye progenitors into optic vesicles (England et al., 2006; Rembold et al., 2006; Martinez-Morales et al., 2009; Picker et al., 2009; Kwan et al., 2012; Ivanovitch et al., 2013). The specification of eye field within the ANP occurs at early stages of CNS formation concomitantly with the specification of the neighboring telencephalic and hypothalamic fields. Observations in fish embryos indicated that patterning acquisition is associated with a profound cell reorganization during which the cells belonging to different domains follow radically different trajectories: telencephalic precursors converge dorsally and medially, whereas eye field cells evaginate medio-laterally (England et al., 2006; Rembold et al., 2006). A notable aspect of this reorganization is the high cohesion with which the eye field cells move, remaining strictly segregated from those of surrounding domains. Domains' separation is controlled by sets of cell adhesion molecules, such as Nlcam, expressed at high levels only in the telencephalic domain, so that up-regulating Nlcam levels in retinal progenitors force them to take trajectories comparable to those of telencephalic cells (Brown et al., 2010). On the other hand, Eph receptors and their Ephrin ligands, expressed in complementary domain in the ANP, promote the active segregation of eye field cells from those of the neighboring domains. Interference with Eph/Ephrin activity results in inefficient segregation of eye and telencephalic cells, with a consequent defective optic vesicle evagination and brain morphogenesis (Cavodeassi et al., 2013). Hence, it seems that the activation of a bidirectional signal upon Eph/Ephrin interaction results in cell repulsion, creating a virtual “fence” between the eye field and the neighboring domains, much resembling Eph/Ephrin role in hindbrain segmentation (Calzolari et al., 2014). The combined functions of the chemokine receptor Cxcr4 and the Wnt signaling pathway instead contribute to maintain cell cohesion within the eye field (Cavodeassi et al., 2005, 2013; Bielen and Houart, 2012). In the absence of *cxcr4*, which is normally expressed in the eye field, telencephalic and eye progenitors intermingle, thereby compromising forebrain morphogenesis (Bielen and Houart, 2012). Similar results have been obtained with the manipulation of components of Wnt non-canonical signaling in the ANP that results in defective cell arrangement during optic vesicle evagination (Cavodeassi et al., 2005).

**Figure 1:**
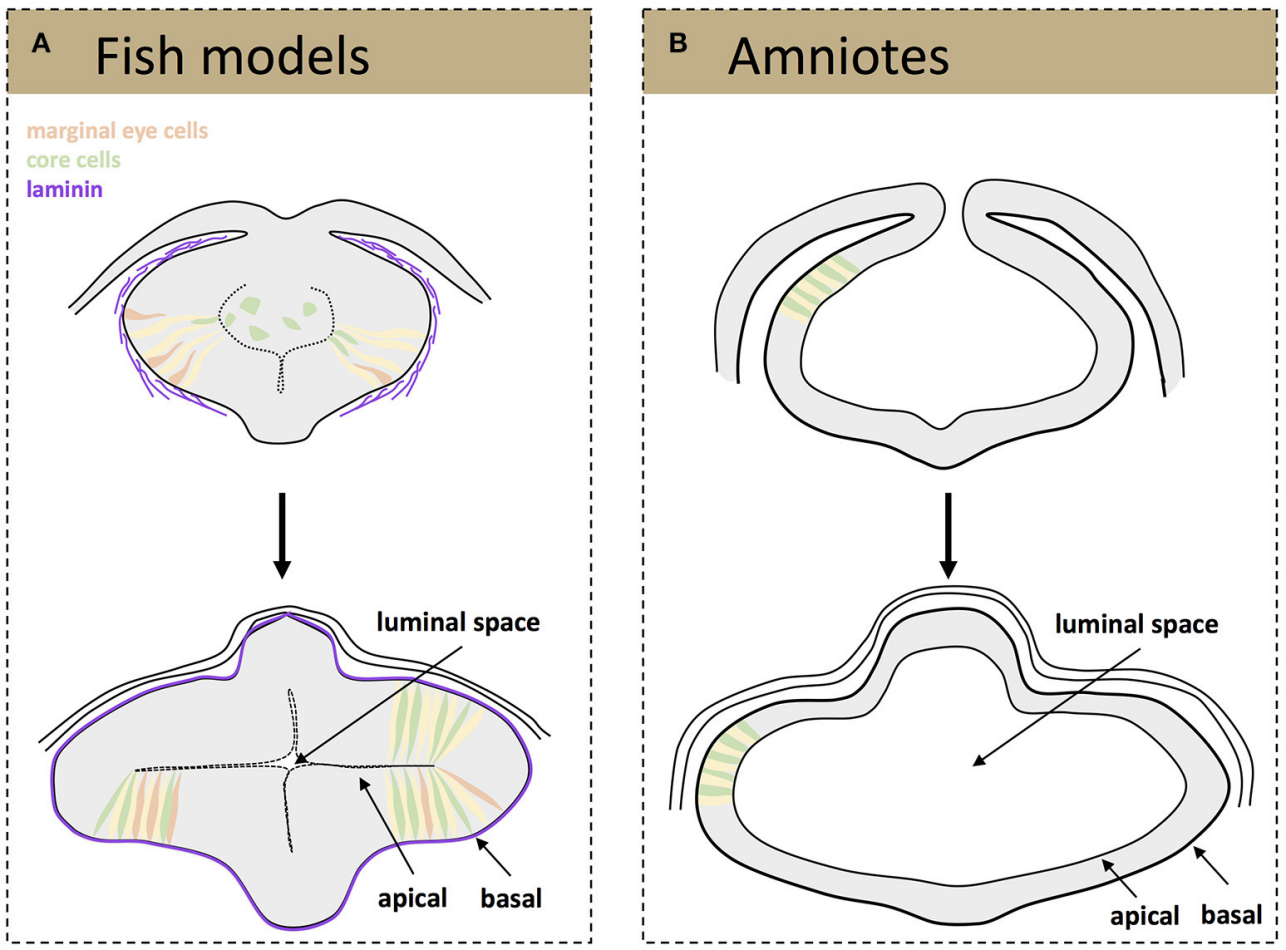
Schematic representation of tissue organization during optic vesicle evagination. Drawings represent frontal sections of the forebrain at the level of the developing optic vesicle from a teleost **(A)** and amniotes **(B)** embryo. (**A**, top) A laminin-rich basement membrane becomes assembled around the fish eye primordium (purple) at the onset of optic vesicle evagination, a process that is thought to promote the elongation and polarization of marginal eye cells (orange, elongating cells; yellow, cells that have already acquired a neuroepithelial appearance). The cells at the core of the eye primordium (green) gradually incorporate into the nascent neuroepithelium by intercalating amongst the marginal cells. (**A**, bottom) Once evagination has concluded the optic vesicles are organized as a neuroepithelial structure, surrounded by a mature basement membrane and with a small luminal space toward the center of the primordia. **(B)** Eye precursors in amniotes are already organized as a mature, cuboidal epithelium at the onset of evagination (top); the optic pits expand and give rise to two optic vesicles with a big luminal space (bottom).

Besides these specific movements, teleost eye field cells undergo dynamic cell shape changes as they organize into optic vesicles. At neural plate stage, eye field cells have a mesenchymal and non-polarized appearance and form a multi-layered structure (Ciruna et al., 2006; Tawk et al., 2007; Ivanovitch et al., 2013). At the onset of zebrafish optic vesicle evagination, cells located at the most lateral (marginal) regions of the eye field elongate and polarize, thus acquiring neuroepithelial characteristics (Figure 1A). This process requires the deposition of a laminin-rich extracellular matrix (ECM) around the eye field. In the absence of laminin, marginal cells do not elongate appropriately and often show disrupted apico-basal organization, failing to become organized into a nascent pseudostratified epithelium (Ivanovitch et al., 2013). This lack of polarity is quite persistent as it has been observed in laminin mutants analyzed and also at optic cup stages (Bryan et al., 2016). As marginal cells are displaced laterally, forming two bulges at the side of the neural tube, the remaining progenitors located at the core of the eye field undergo the same elongation and polarization to intercalate among the lateral ones till the end of the evagination process (Figure 1A; Ivanovitch et al., 2013). The net result is a dynamic expansion of the optic vesicles (Rembold et al., 2006; Kwan et al., 2012; Ivanovitch et al., 2013) although the underlying molecular control is still poorly understood. One of the possible regulators of this cell intercalation might be the non-canonical Wnt signaling pathway, as its activation drives similar cell rearrangements in other morphogenetic processes, such as the convergence-extension rearrangements occurring during embryonic gastrulation (Heisenberg et al., 2000). Other signaling pathways, such as those triggered by Fgf and Hh ligands and involved in partitioning of the nascent optic vesicle along the proximo-distal and naso-temporal axis (Picker and Brand, 2005; Picker et al., 2009; Hernandez-Bejarano et al., 2015), might be also relevant for these early morphogenetic movements, as morphogenesis and patterning are tightly coordinated events (Picker et al., 2009).

Independent of their molecular regulation, the end result of the early morphogenetic movements occurring in the teleost neural plate is the formation of two bulges composed of a folded neuroepithelium separated by a virtual ventricle (Figure 1A). One of the outstanding questions is whether the cell rearrangements observed during the reorganization of the teleost eye field into optic vesicles occur also in birds and in mammals. The difficulties in obtaining homogeneous labeling of bird eye field cells, a precondition for specific imaging, and the *in utero* development of mammalian embryos have so far hindered comparative studies at these very early stages. Nevertheless, the sequence of events or their timings are likely different, although optic vesicle evagination in chick embryos and mammal organoids requires the presence of a laminin-rich ECM (Svoboda and O'Shea, 1987; Eiraku et al., 2011). Indeed, the incipient optic vesicles of chick and mouse embryos are composed of already polarized neuroepithelial cells of a cuboidal shape that appear to elongate as the optic vesicle evaginate (Figure 1B) to shorten again when the optic vesicle is fully formed (Camatini and Ranzi, 1976; Svoboda and O'Shea, 1987). Unlike teleosts, the mammalian and bird eye neuroepithelium surrounds a large ventricle and its enlargement likely occurs owing to the incorporation of already polarized neuroepithelial cells coming from the adjacent and already folded neural tube (Figure 1B). Nevertheless, future technical advances are needed to verify if this assumption is correct.

## Bending the optic vesicles into optic cups

**Figure 2:**
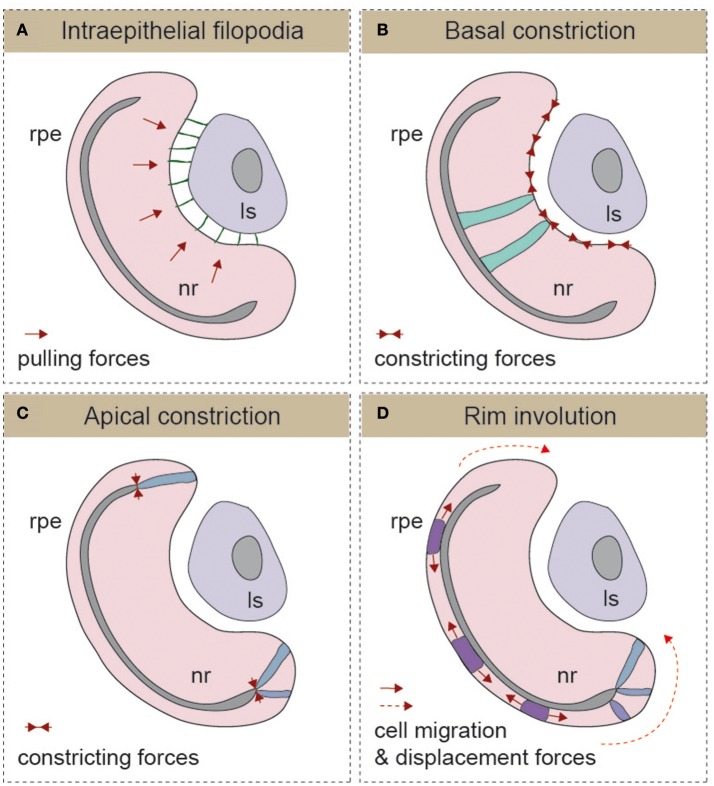
Schematic representation of the different mechanisms described for optic cup morphogenesis **(A)** Intraepithelial filopodia, **(B)** Basal constriction, **(C)** Apical constriction, and **(D)** Rim involution mechanisms are represented. In each one of the panels, the direction of morphogenetic forces is indicated with solid red arrows and cells displacement trajectories with dotted red arrows. The morphology of representative cells is also depicted. Ls, lens; nr, neural retina; rpe, retinal pigmented epithelium.

From the previous paragraphs, it seems reasonable to state that early eye morphogenesis is not equivalent among vertebrates. However, there is a developmental window in which embryos of the same animal group but of different species display highest anatomical similarity (Slack et al., 1993). This reference concept is known as the “phylotypic period” in the “Evo-Devo” field, and for eye development, it arguably corresponds with the early stages of optic cup formation. It is within this window that the morphology of the eye rudiment converges in a common architecture for all vertebrate species (Plouhinec et al., 2005). It is also during this period—after eye precursors get specified in the anterior neural tissue, but before neuronal differentiation begins—that the basic blueprint of the organ is established. This is achieved through the segregation of conserved gene regulatory circuits conferring identity to each one of the presumptive territories of the optic cup: the neural retina, the retinal pigmented epithelium (RPE), and the optic stalk (Fuhrmann, 2010). Gene regulatory networks specific for ocular domains are, in turn, under the control of polarizing morphogens such as FGFs (secreted from the presumptive lens and the retina), Shh and nodal (secreted from the CNS midline), and activins, Wnts, and BMPs (secreted by the extraocular mesenchyme and the dorsal ectoderm; Adler and Canto-Soler, 2007; Martinez-Morales and Wittbrodt, 2009; Fuhrmann, 2010; Steinfeld et al., 2013). In spite of the divergent mechanisms responsible for the formation of the optic vesicle in different vertebrate groups, their final organization in all species is that of a pseudostratified epithelium in which precursor cells are elongated and polarized (Ivanovitch et al., 2013; Strzyz et al., 2016). Regardless of the vertebrate group considered and the size of the separating ventricle, the embryonic vesicle consists of two epithelial layers that oppose apically and that will bend wrapping around the lens vesicle as development proceeds. How this bending occurs, is the eye morphogenetic event that has perhaps received the most attention in recent years, leading to significant advances in our understanding of its cellular and molecular bases. The outcome, to which different groups and model organisms have contributed, is the identification of a number of morphogenetic movements and cell shape changes that are outlined in the following paragraphs (Figure 2), underscoring their possible discrepancies.

### Folding through lens-retina coordination

The formation of the optic cup and the invagination of the lens vesicle occur simultaneously. Therefore, in principle, both processes could be linked, and there are studies showing that optic vesicle development depends on early inductive signals emanating from the pre-lens ectoderm (Hyer et al., 2003). Furthermore, in chick embryos, early removal of the lens ectoderm or degradation of the corresponding ECM prevents optic vesicle in-folding (Oltean et al., 2016). In mouse embryos, there is also evidence of the presence of lens-derived filopodia-dependent pulling forces connecting the lens ectoderm and the optic vesicle neuroepithelium (Figure 2A) (Chauhan et al., 2015). Genetic interference with filopodia formation, by conditional ablation of the Rho GTPase family member *Cdc42* or of its *IRSp53* effector in the lens, results in an uncoordinated folding of both lens and retinal epithelial surfaces (Chauhan et al., 2009). The presence of inter-epithelial filopodia is likely a mammalian or a species-specific phenomenon, as these types of extensions are apparently absent in many of the other vertebrate systems studied (i.e., zebrafish and chicken). Nevertheless, filopodia emerging from basal surface of the neural retina have been described in medaka (Martinez-Morales et al., 2009; Porazinski et al., 2015), but their contribution to the folding of the optic cup has not been investigated. This allows inferring the existence of alternative uncharacterized mechanisms responsible for the coordinated adjustment of the optic cup and lens morphology in vertebrates. Furthermore, there is evidence indicating that the presence of the lens vesicle is not an absolute requirement for optic cup folding. Surgical removal of the lens shortly after invagination starts (i.e., HH13) does not impair optic cup folding in chick embryos (Oltean et al., 2016). Similar conclusions can be drawn from the induction of ectopic cups by *six3* overexpression in medaka embryos (Loosli et al., 1999) and the generation of self-organized eyes from ES-cultured cells in mammals (Eiraku et al., 2011; Nakano et al., 2012). In both cases, optic cup morphogenesis proceeds in the absence of a lens, suggesting that the acquisition of a cup shape is an intrinsic property of the organoid tissue. Lens-retinal interaction has, however, other functions. For example, the physiology of vision imposes the existence of fine-tuning mechanisms to adjust the inter-epithelial distance and the curvature of the lens to the photoreceptive surface of the retina and vice-versa.

### Folding by basal constriction and ECM-attachment

Optic cup formation entails the active bending of the neuroepithelium toward its basal surface (Figure 2B). By analogy to apical constriction processes described in other epithelia (Sawyer et al., 2010), basal constriction of the neuroblasts' end-feet was hypothesized as a main morphogenetic mechanism for retinal bending (Martinez-Morales and Wittbrodt, 2009; Martinez-Morales et al., 2009). This initial suggestion was based on the observation of wider basal feet in the retinal epithelium of medaka *opo* mutants, which are characterized by strong optic folding defects (Martinez-Morales et al., 2009). The existence of a basal shrinkage process was, however, formally confirmed only very recently via direct imaging analysis of the neuroblasts' behavior in zebrafish (Figure 2B) (Nicolas-Perez et al., 2016; Sidhaye and Norden, 2017). As reported for other constricting epithelia (Martin et al., 2009; Gorfinkiel and Blanchard, 2011), zebrafish retinal precursors exhibit a pulsatile behavior with episodic contractions of the basal feet that correlate with myosin foci condensation (Nicolas-Perez et al., 2016). A number of reports indicate that proper attachment to the extracellular matrix is essential for the transmission of the constriction forces that shape the organ. The direct role of integrins in the formation of the eye chamber has been documented in teleosts, chicken, and human tissues (Svennevik and Linser, 1993; Martinez-Morales and Wittbrodt, 2009; Nakano et al., 2012). Furthermore, it has been shown that the polarized trafficking of integrin receptors toward the basal surface—controlled by the molecular antagonism between the protein Opo (*ojoplano*) and the clathrin adaptors numb and numb-like—plays an essential role during optic cup folding (Bogdanovic et al., 2012). A critical morphogenetic role for the ECM is also supported by the ocular phenotype of laminin mutants in zebrafish including *lama1* and *lamc1* mutants, which show optic cup defects and impaired constriction of the basal end-feet of neuroepithelial cells (Lee and Gross, 2007; Bryan et al., 2016; Nicolas-Perez et al., 2016; Sidhaye and Norden, 2017).

Experimental measurement of tissue stiffness in folding optic cups in chick embryos suggests that the ECM constrains basally the tangential growth of the epithelium, preventing its natural evagination and forcing tissue to infold (Oltean et al., 2016). Computational modeling based on data obtained from collagenase treated cups predicts that the ECM-provided constrain is sufficient to drive the morphogenesis of the organ (Oltean et al., 2016). However, laser-induced local ablation of the basal surface in the invaginating retina of the zebrafish results in a fast relaxation of the entire cup toward its basal side (Nicolas-Perez et al., 2016). These apparently conflicting observations would indicate a complex mechanical contribution of the ECM to optic cup morphogenesis. The precise balance between the ECM biophysical properties—as a constraining barrier for growth and as a belt for the transmission of tensional forces at a supra-cellular scale—may critically depend on the developmental window and the model organism considered and might need further studies.

### Folding by apical constriction and proliferation control of the optic cup hinge

The bi-layered structure of the optic primordium imposes that those cells positioned at the hinge region—between the inner/medial, future retina, and outer/lateral future RPE layers—have to constrict apically to allow acute bending at the tissue margin (Figure 2C). This has set forward the idea that the constriction of these hinge cells may contribute actively to the invagination process. The observation of changes in cell shape and in the levels of phosphorylated myosin light chain in mouse ESCs-derived, and even with human ES cells (Nakano et al., 2012), organoids provided the ground for a spring-based computational model of optic cup formation (Eiraku et al., 2011, 2012). According to this “relaxation-expansion” hypothesis, the combination of tangential expansion of the epithelium and apical constriction at the rims together with a differential stiffness of the outer and inner optic cup layers suffices for an epithelial fold-back allowing for invagination of the optic cup (Eiraku et al., 2012). A variation on this idea has come from studies in mouse embryos. Preventing the secretion of all Wnt ligands from the placodal ectoderm of the lens reduces the number of cells at the outer hinge layer and interferes with complete folding of the optic cup (Carpenter et al., 2015). These same experiments, however, suggest that differential forces at the hinge are not required for the formation of the cup, as a part of the folding nevertheless occurs. One possibility is that the entire outer layer of the optic cup (and not only that at the rim) might be important for folding. Indeed, in Otx mouse mutants, in which the RPE is specified as a neural retina, the optic cup does not fold properly (Martinez-Morales et al., 2001). Alternatively, basal constriction of inner layer cells together with changes at the hinge could synergize to induce folding. Nevertheless, at the moment, there is still insufficient experimental data to validate the predictions of the “relaxation-expansion” model that, although attractive, needs to be tested in living embryos.

### Rim involution as a motor for optic cup folding

A number of studies in zebrafish have described that, during optic cup invagination, precursor cells translocate from the outer/medial epithelial layer (presumptive RPE) to the inner/lateral epithelial layer (presumptive neural retina; Figure 2D). Initial observations, combining toluidine staining and fluorescent tracking in zebrafish embryos, reported a progressive narrowing of the outer layer at the expenses of the inner layer (Li et al., 2000). This study shows that approximately the peripheral third of the 24 hpf neural retina derives from outer layer precursors. More recently, time-lapse analyses of the invagination stages (18–24 hpf) confirmed previous observations and provided a detailed description of the translocation movements *in vivo* (Picker et al., 2009; Kwan et al., 2012). A subsequent imaging study showed that this phenomenon has a ventral and temporal prevalence, depends on BMP signaling, and is relevant for optic cup morphogenesis (Heermann et al., 2015). Indeed, progenitors flowing through the rim may act as a mechanism for coupling morphogenesis and retinal determination, with flattening of the presumptive RPE acting as a motor of the translocation process (Heermann et al., 2015). The active migratory behavior of the rim has been recently further analyzed, thereby leading to somewhat different conclusions. In this case, the interaction of basal lamellipodia with the underlying ECM directs the progenitors to flow through the rim region (Sidhaye and Norden, 2017). Therefore, cell-ECM interactions would be not only essential for basal constriction but also for active rim migration/involution (Nicolas-Perez et al., 2016; Sidhaye and Norden, 2017). To what extent the both morphogenetic mechanisms—RPE flattening and collective migration at the rim—are acting in a coordinated manner, in isolation, or together with constriction mechanisms remains to be investigated. It is also important to consider that although some specific morphogenetic movements observed during optic cup formation in zebrafish (i.e., pinwheel and anterior rotation) have also been confirmed in chicken, this is not the case for rim involution movements (Kwan et al., 2012). Therefore, whether rim involution movements represent a universal mechanism for optic cup folding in vertebrates or a species-specific adaptation to the fast zebrafish development is still uncertain.

In conclusion, there is no doubt that eye formation requires cell rearrangements perhaps beyond what we could have previously envisioned, but it is also clear that many questions still remain open. Are these morphogenetic mechanisms, mostly described for teleost fish, universal in all vertebrates? Which is their relative contribution in each species? Do they act coordinately, sequentially, or in an independent manner? What are the driving forces for each one of the cell rearrangement described so far? Tackling all these complex questions will require further comparative analyses and comprehensive computational modeling. Furthermore, we know very little on how tensional forces and cell shape changes integrate with instructive signaling and tissue patterning mechanisms to guarantee the precise 3D, and even 4D, architecture of the vertebrate eye. The latter is an additional challenging question that needs to be solved in future studies.

## Author contributions

All authors listed, have made substantial, direct and intellectual contribution to the work, and approved it for publication.

### Conflict of interest statement

The authors declare that the research was conducted in the absence of any commercial or financial relationships that could be construed as a potential conflict of interest.
